# Recent Progress in Fabrication of Antimony/Bismuth Chalcohalides for Lead-Free Solar Cell Applications

**DOI:** 10.3390/nano10112284

**Published:** 2020-11-18

**Authors:** Yong Chan Choi, Kang-Won Jung

**Affiliations:** Division of Energy Technology, Daegu Gyeongbuk Institute of Science & Technology (DGIST), Daegu 42988, Korea; kw.jung@dgist.ac.kr

**Keywords:** antimony chalcohalides, bismuth chalcohalides, solar cells

## Abstract

Despite their comparable performance to commercial solar systems, lead-based perovskite (Pb-perovskite) solar cells exhibit limitations including Pb toxicity and instability for industrial applications. To address these issues, two types of Pb-free materials have been proposed as alternatives to Pb-perovskite: perovskite-based and non-perovskite-based materials. In this review, we summarize the recent progress on solar cells based on antimony/bismuth (Sb/Bi) chalcohalides, representing Sb/Bi non-perovskite semiconductors containing chalcogenides and halides. Two types of ternary and quaternary chalcohalides are described, with their classification predicated on the fabrication method. We also highlight their utility as interfacial layers for improving other solar cells. This review provides clues for improving the performances of devices and design of multifunctional solar systems.

## 1. Introduction

Since the Snaith group reported the 10.9% milestone power conversion efficiency (PCE) required for industrial applications for lead-based perovskite (Pb-perovskite) solar cells in 2012 [1], many types of Pb-perovskite solar cells have been fabricated, with performance significantly improving over the past few years [2,3,4,5,6,7]. At present, the certified PCE exceeds 25% [8], approaching the theoretical maximum efficiency for multi-junction Pb-perovskite solar cells [9]. This efficiency is also comparable to those of commercial solar systems based on Si, CdTe, and Cu(In,Ga)Se_2_. Moreover, high-performance Pb-perovskite solar cells can be manufactured through solution processing at a low temperature of <150 °C, which can reduce costs. Therefore, these characteristics make them the most promising alternative to current photovoltaic systems. However, Pb-perovskite solar cells exhibit limitations for commercialization, with potential health problems and stability being the two main barriers [6,7,10,11,12,13,14,15]. In particular, Pb can be easily released from the Pb-perovskite because of its instability, which can cause major health problems [10,11,12,13,14,15]. Although techniques of material and interface engineering, surface passivation, and encapsulation can significantly improve the stability of Pb-perovskite [7,15,16,17], thereby minimizing the Pb loss, the persistent toxicity problem requires attention to enhance commercialization.

To address these issues, many researchers have focused on finding Pb-free and stable materials with comparable optoelectronic properties. The Pb-free photovoltaic materials proposed as alternatives to date are presented in Table 1. Replacing Pb by tin (Sn) or germanium (Ge), with similar ionic radius and belonging to the same group of the periodic table, in Pb-perovskites is a simple method for fabricating Pb-free materials while maintaining the perovskite structure. These materials are known as Pb-free perovskites. In particular, Sn-based perovskites ASnX_3_ (A = Cs^+^, organic cations; X = Cl, I, and Br) exhibit properties comparable to those of Pb-perovskites such as optimal band gaps (*E*gs) of 1.1–1.4 eV, high carrier mobilities, long carrier lifetimes, and long diffusion lengths [10,11]. Thus, many researchers have devoted attention to developing Sn-based perovskite solar cells [10,11]. Consequently, a record PCE of 11.4% was achieved through the FASnI_3_ (FA = CH_5_N_2_^+^) solar cell by introducing a phenylhydrazine hydrochloride [18]. However, Sn-perovskites still involve the serious disadvantage of rapid decomposition because Sn is readily oxidized from the +2 to +4 state on exposure to air [10,11]. Another approach for fabricating Pb-free perovskites involves replacing two Pb^2+^ ions with ions of two metals with oxidation states of +1 and +3 to form double perovskites represented as A_2_M^I^M^III^X_6_ [12,19], with the Cs_2_AgBiBr_6_ as a typical example. Alternatively, the two Pb^2+^ ions are replaced by a tetravalent metal ion, such as Sn^4+^ or Ti^4+^, forming compounds with the general formula A_2_M^IV^X_6_ [12,20]. Such compounds are termed vacancy-ordered double perovskites, with the Cs_2_SnI_6_ as a prime example. In addition, two-dimensional (2D) perovskites $A_{3}M_{2}^{\mathrm{III}}X_{9}$ are produced by replacing the Pb^2+^ ions with trivalent metal ions such as Sb^3+^ or Bi^3+^ [15,21]. Although these Pb-free double and 2D perovskites display significant stability improvement over Pb- and Sn-perovskites, efficiency remains a limitation.

Apart from these Pb-free perovskites, antimony/bismuth (Sb/Bi)-based non-perovskites are another alternative to Pb-perovskites. Unlike perovskites, most of these non-perovskites crystallize in a layered structure, with the layers linked by weak van der Waals forces. This anisotropic crystal structure provides unique and interesting properties that can significantly affect photovoltaic performance [25,26,27]. To date, many Sb/Bi non-perovskites for solar cells have been reported, and these comprise two types, according to elemental composition. The first type is the Sb chalcogenides involving an orthorhombic structure, such as the Sb_2_Ch_3_ and CuSbCh_2_ (Ch = S, Se). In fact, studies on these as photovoltaic materials predates those of Pb-perovskites because of their promising properties, such as the tunable *E*g values of 1.0–1.8 eV, high visible light absorption coefficient, stability, low toxicity, and earth-abundant constituents [25,26]. Although varied engineering methods and device architectures have been employed to achieve high-efficiency for Sb chalcogenide solar cells, the performances of these cells remained below the 10% milestone until 2018 [26,27,28,29,30,31,32,33,34,35]. However, recently, a PCE of 9.2% was obtained from the [001]-oriented Sb_2_Se_3_ nanorod solar cells [36], and finally, a PCE of 10.5% was reported by Chen’s group from the hydrothermally deposited Sb_2_(S,Se)_3_ thin film solar cells [22,37]. 

Sb/Bi chalcohalides represent the other type of Sb/Bi non-perovskites, comprising Sb/Bi-based semiconductors containing halides and chalcogenides. Following the initial application of Sb sulfoiodide (SbSI) in solar cells by the Seok group in 2018 [38], multiple Sb/Bi chalcohalide solar cells have been proposed. Thus far, the materials investigated for use in solar cells include ternary (MChX and M_13_Ch_18_X_2_, where M = Sb, Bi) [23,38,39,40,41,42,43,44,45,46,47,48,49,50,51,52,53] and quaternary chalcohalides ($M_{2}^{\mathrm{II}}M^{\mathrm{III}}{\mathrm{Ch}}_{2}X_{3}$, where M^II^ = Sn, Pb; M^III^ = Sb, Bi) [24,54]. These chalcohalides commonly exhibit advantageous properties that can be adjusted for use in solar cells. In particular, the electronic structure of the most studied MChX family is similar to that of Pb-perovskites, with beneficial properties for solar cells such as high dielectric constant, low effective mass, and tunable *E*g [39,42,43,44,55]. Therefore, high-performance MChX solar cells comparable to Pb-perovskite cells are expected due to these properties. Recently, the Seok group reported a PCE of 4.07% for Sb_0.67_Bi_0.33_SI solar cells, highlighting the high-efficiency potential for the MChX family [23]. In addition, the MChX family is suitable for other applications including the fabrication of room-temperature radiation detectors and p-type transparent conductors [39]. This wide-ranging applicability facilitates designing multifunctional devices. In addition to the MChX family, PCEs of 0.85% and 4.04% have been reported for solar cells based on M_13_Ch_18_X_2_ and $M_{2}^{\mathrm{II}}M^{\mathrm{III}}{\mathrm{Ch}}_{2}X_{3}$, respectively. However, the highest PCE achieved for Sb/Bi chalcohalide solar cells remains at around 4%, although the performance has significantly improved over the past few years. 

Here, we focus on Sb/Bi-based chalcohalides, including emerging solar material such as MChX compounds, since this type of non-perovskites lack a comprehensive review. Therefore, an up-to-date review summarizing the rapid development of Sb/Bi chalcohalide solar cells and highlighting future research directions is necessary. In this review, we aim to summarize the advances in Sb/Bi chalcohalide solar cells research. To this end, we briefly introduce the crystal and energy band structures of Sb/Bi chalcohalides. Then, we classify these materials based on the fabrication method and discuss their photovoltaic performances. Furthermore, we highlight their usage as interfacial layers for enhancing solar cells. This review presents a step toward the production of high-performance Pb-free non-perovskite chalcohalide solar cells. Note that we have excluded perovskite-based chalcohalide such as (CH_3_NH_3_)SbSI_2_ [56] from this review. 

## 2. Crystal and Energy Band Structures of Sb/Bi Chalcohalides

In this section, the crystal and energy band structures of Sb/Bi chalcohalides used to date for solar cells are briefly presented. Depending on the number of elements and composition, Sb/Bi chalcohalides with different structures can be created, as shown in Table 2. The ternary chalcohalides employed for solar cells are the MChX and M_13_Ch_18_X_2_ types. The MChX type, such as SbSI and BiSI, involves the orthorhombic structure with the *Pnma* space group, crystallizing into an [(MChX)_2_]*_n_* double-chained structure, with the adjacent chains joined by van der Waals forces [57,58]. Conversely, the M_13_Ch_18_X_2_ type such as the Bi_3_S_18_I_2_ possesses a hexagonal structure with a ribbon-shaped (M_4_Ch_6_)_∞_ subunit. The M_4_Ch_6_ subunits form six spokes around the central hexagonal channel at the corners of the unit cell, with iodine in between [53,58]. For the quaternary chalcohalides ($M_{2}^{\mathrm{II}}M^{\mathrm{III}}{\mathrm{Ch}}_{2}X_{3}$), such as Pb_2_SbS_2_I_3_ and Sn_2_SbS_2_I_3_, crystallization produces the orthorhombic structure with the *Cmcm* space group [24,54,59]. 

**Figure 1:**
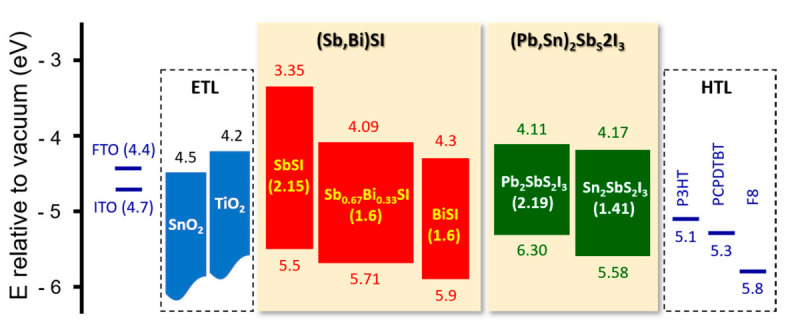
Energy band diagram of typical Sb/Bi chalcohalides. The SbSI, Sb_0.67_Bi_0.33_SI, BiSI, Pb_2_SbS_2_I_3_, and Sn_2_SbS_2_I_3_ energy levels were obtained from [23,38,47,54] and [24], respectively. For comparison, the energy levels for typical conducting oxides (F-doped SnO_2_ (FTO) and In-doped SnO_2_ (ITO)), the electron transporting layer (ETL), and hole transporting layer (HTL) are included. P3HT, PCPDTBT, and F8 denote poly(3-hexylthiophene), poly[2,6-(4,4-bis(2-ethylhexyl)-4*H*-cyclopenta[2,1-b;3,4-b’]dithiophene)-alt-4,7-(2,1,3-benzothiadiazole)], and poly(9,9-di-n-octylfluorenyl-2,7-diyl), respectively.

To employ Sb/Bi chalcohalides in solar cells, the energy band structure deserves priority because of its importance in light harvesting and conversion. Specifically, the *E*g should be checked because it determines the maximum PCE achievable for each material according to the Shockley–Queisser limit [60,61]. Thus, materials with an *E*g value between 1.10 and 1.55 eV are preferred for solar cells. Figure 1 displays the energy band diagram of typical Sb/Bi chalcohalides reported to date. The positions of the conduction band minimum and valence band maximum as well as the *E*g value vary depending on the elemental composition and number of elements. Along with the chalcohalides shown in Figure 1, Sb/Bi chalcohalides exhibit *E*g values varying from 0.75 eV for Bi_13_S_18_I_2_ [53] to 2.31 eV for SbSBr [42]. These results indicate that their band structures can be tuned via chemical substitution, and that the electron transporting layer (ETL) and hole transporting layer (HTL) applications necessitate selectivity for each solar cell depending on the chalcohalide used. In addition to the band structures, other factors such as the optical absorption strength, charge effective mass, dielectric constant, and defects require consideration [44,61]. However, research on these remains insufficient, and this highlights the need for further studies. 

## 3. Theoretical Insights on Sb/Bi Chalcohalides as Solar Absorbers

Theoretical calculations, such as first-principle methods, provide further insight into the potential of specific materials (e.g., as solar absorbers) and clues for designing device structures. However, the research on such theoretical investigations is very limited because Sb/Bi chalcohalide solar cells are still in their early stages of development compared to the Pb-perovskite cells. Thus, in this section, theoretical insights into only the most studied MChX family are briefly introduced.

Based on the first-principle calculations, Brandt et al. identified the MChX family as promising solar absorbers due to its low effective masses, large dielectric constants, and strong absorption, as shown in Table 3 [62]. They further found that BiSI and BiSeI are most suitable for achieving high-performance solar cells because of their much stronger spin-orbit coupling. The suitability of these Bi compounds for solar cells was also confirmed by other groups [39,43,44,63]. Ganose et al. suggested that the conducting oxide and HTL should be selected for efficient charge transfers by considering the electron affinity (EA = 4.9–5.0 eV) and ionized potential (IP = 6.2–6.4 eV) of these Bi chalcohalides, respectively [43]. They also concluded from the defect analysis that these Bi compounds represent intrinsic semiconductors regardless of fabrication conditions, making them best suited for application in *p-i-n* device architecture [44].

Butler et al. analyzed the band structures of SbChX (SbSI, SbSeI, and SbSBr) by different calculation methods [42,55]. The effective masses were calculated to be below 0.65, indicating that SbChX have high charge carrier mobilities suitable for solar cells. They also found that the SbSBr have deeper IP energy (5.8 eV) than that of I-containing SbChX (5.3 eV for SbSeI and 5.4 eV for SbSI). This different IP energy suggests that contacting layers such as ETL and HTL should be selected depending on the halide ion of SbChX for optimal device performance [42]. For example, the contacting layers used in Cu_2_ZnSnS_4_ (CZTS) can be applied to SbSBr solar cells due to their similar IP value with that of CZTS. In addition, a heterojunction structure composed of SbSI/SbSBr with SbSBr epitaxially grown on SbSI was proposed for efficient charge separation based on their closely matched lattice parameters and band offsets [55].

## 4. Sb/Bi Chalcohalide Solar Cells Fabrication

The fabrication of high-quality materials with adequate morphologies and properties is essential for manufacturing high-performance solar cells. However, methods for producing Sb/Bi chalcohalide solar cells are scant, with those existing lacking the optimization necessary to provide high-efficiency solar cells. Therefore, developing methods to control and optimize the properties of chalcohalides suitable for solar cells is imperative. Sb/Bi chalcohalides used for solar cells are prepared by many techniques including spray pyrolysis [40], spin coating [24,45,46,47,51], solvothermal synthesis [49,53], and mixed techniques [23,38,48,65]. In this section, the fabrication methods reported to date are categorized and described, with the solar cells fabricated presented by the method in Table 4.

### 4.1. One-Step Deposition

**Figure 2:**
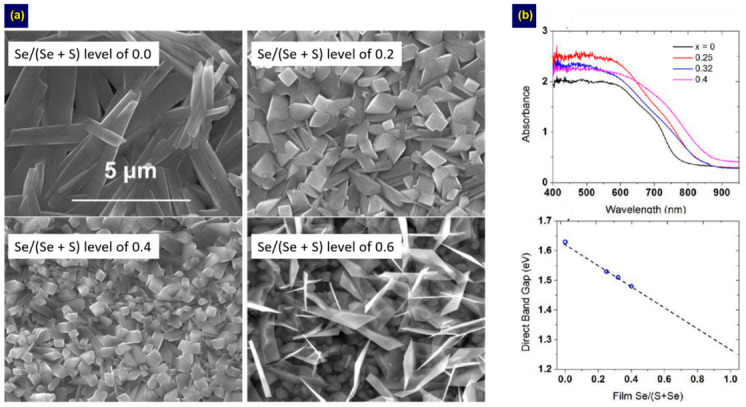
Images and plots characterizing Se-doped BiSI films fabricated by spray pyrolysis at varied Se doping levels including: (**a**) surface morphologies; (**b**) absorption and direct *E*g graph. Adapted with permission from *J. Phys. Chem. C* **2012**, *116*, 24878–24886. Copyright 2012 American Chemical Society [40].

In the one-step method, Sb/Bi chalcohalides are directly deposited using a precursor solution by the spray or spin-coating techniques. Hahn et al. deposited Se-doped BiSI films by spraying a precursor solution on a pre-heated F-doped SnO_2_ (FTO) substrate at 275 °C [40]. The Se doping levels were controlled by adjusting the concentration of thiourea (TU) and SeO_2_ in the precursor solution. They found that the morphology changed from microscale rods to cube-like structures as the Se amount increased (Figure 2a). The optical *E*g decreased linearly with increasing Se content, as shown in Figure 2b. Then, the researchers applied these Bi(S,Se)I films for solar cell fabrication, obtaining a PCE of 0.012% for an FTO/Pt/CuSCN/BiSI/FTO device.

**Figure 3:**
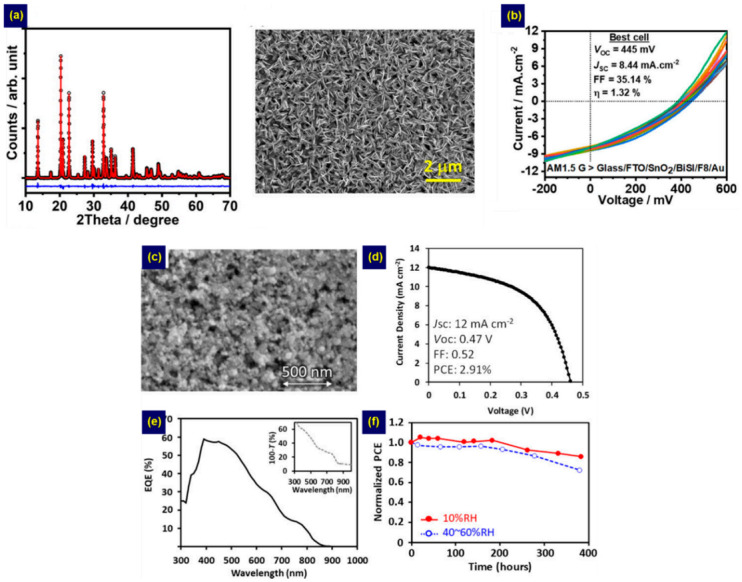
Images and plots for Sb/Bi chalcohalides fabricated by the one-step method based on the spin-coating technique showing: (**a**) structure and surface morphology; (**b**) photovoltaic device performance for BiSI films fabricated by Tiwari et al. [46]. Adapted with permission from *ACS Appl. Energy Mater.* **2019**, *2*, 3878–3885. Copyright 2019 American Chemical Society [46]. (**c**) Surface morphology image of Sb_2_S_3_-containing SbSI; (**d**–**f**) the device performance. Adapted with permission from *Chem. Mater.* **2020**, *32*, 6416–6424. Copyright 2020 American Chemical Society [51].

Recently, Tiwari et al. applied the spin coating technique to the one-step method in fabricating BiSI films [46]. They used a molecular solution synthesized by dissolving Bi(NO_3_)_2_·5H_2_O, TU, and NH_4_I in a 2-methoxyethanol and acetylacetone mixture for the spin coating. Using this method, flake-shaped BiSI films were produced (Figure 3). To apply these films to solar cells, they used SnO_2_ and F8 as the ETL and HTL, respectively, obtaining a PCE of 1.32% for an Au/F8/BiSI/SnO_2_/FTO device (Figure 3b). Similarly, Nishikudo et al. used an Sb(EtX)_3_ single crystal for a spin coating based on the one-step method [51]. To fabricate SbSI solar cells, the solution, synthesized by dissolving the Sb(EtI)_3_ single crystal and SbI_3_ in dimethyl sulfoxide, was spin-coated onto a mesoporous TiO_2_ (mp-TiO_2_)/TiO_2_ blocking layer (TiO_2_-BL)/FTO substrate and annealed at 200–240 °C. Then, the HTL and Au were sequentially deposited. The Sb_2_S_3_-containing SbSI structure obtained at 240 °C exhibited better device performance than that with the SbSI. Furthermore, thiophene-containing HTL such as the poly[2,6-(4,4-bis(2-ethylhexyl)-4*H*-cyclopenta[2,1-b;3,4-b’]dithiophene)-alt-4,7-(2,1,3-benzothiadiazole) (PCPDTBT) and poly(3-hexylthiophene) (P3HT) was reported to significantly contribute to improving device performance. As a result, they obtained an impressive PCE of 2.91% from the Sb_2_S_3_-containing SbSI device involving the PCPDTBT HTL, and the device showed good stability under high humidity (Figure 3c–f). In addition to ternary MChX, the one-step spin-coating method is usable in fabricating quaternary chalcohalides ($M_{2}^{\mathrm{II}}M^{\mathrm{III}}{\mathrm{Ch}}_{2}X_{3}$). Recently, Nie et al. synthesized a precursor solution by dissolving SbCl_3_, TU, and SnI_2_ in *N*,*N*-dimethylformamide [24]. Then, the solution was spin-coated on mp-TiO_2_/TiO_2_-BL/FTO and annealed to fabricate quaternary Sn_2_SbS_2_I_3_ nanostructures. The as-prepared Sn_2_SbS_2_I_3_ displayed a suitable *E*g of 1.41 eV, while the Sn_2_SbS_2_I_3_ device showed a PCE of 4.04% and good stability against humidity.

### 4.2. Two-Step Deposition Method

In the two-step deposition method, chalcogenides (M_2_Ch_3_) are fabricated (step 1) and then converted into chalcohalides (MChX) through the reaction of M_2_Ch_3_ and MX_3_ (step 2). This reaction is expressed in Equation (1).

M_2_Ch_3_ + MX_3_ → 3MChX, (1)

**Figure 4:**
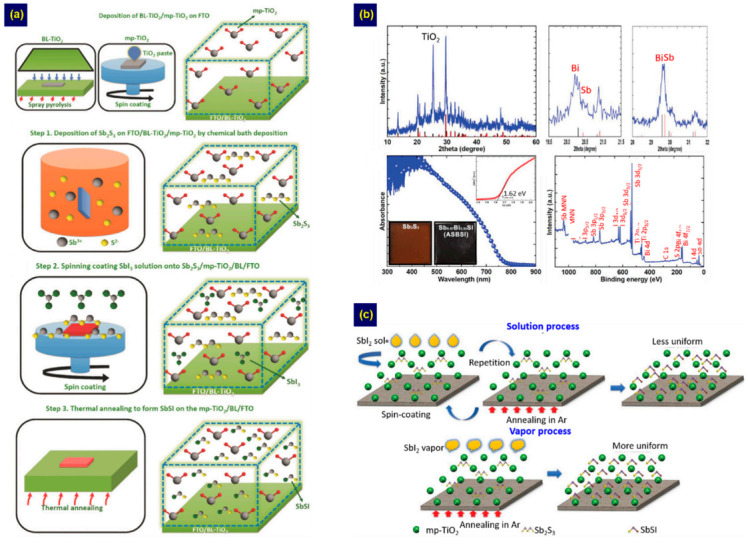
(**a**) Schematic illustration of the two-step method for SbSI fabrication. Adapted from [38], with permission from John Wiley and Sons, 2017; (**b**) Structure, absorption, and X-ray photoelectron spectroscopy properties of the Sb_0.67_Bi_0.33_SI. Adapted from [23], with permission from John Wiley and Sons, 2019; (**c**) Schematic illustration of the two processes utilized in step 2 of the SbSI fabrication. Adapted from [65], with permission from John Wiley and Sons, 2019.

This deposition method was first applied by the Seok group for fabricating SbSI solar cells (Figure 4a) [38]. In step 1, amorphous Sb_2_S_3_ was deposited on an mp-TiO_2_/TiO_2_-BL/FTO substrate by chemical bath deposition (CBD), accompanied by crystallization at 300 °C. Then, the crystalline Sb_2_S_3_ was converted to SbSI by multiple cycles of spin coating with an SbI_3_ solution, followed by annealing (step 2). A PCE of 3.05% was obtained from an Au/PCPDTBT/SbSI/mp-TiO_2_/TiO_2_-BL/FTO solar cell. Furthermore, they fabricated Bi-alloyed SbSI, i.e., Sb_0.67_Bi_0.33_SI, using a BiI_3_ solution instead of SbI_3_ in step 2 (Figure 4b) [23]. This material absorbs more light, producing a higher short-circuit current density because of its narrower *E*g (1.67 eV) than SbSI. Thus, a better PCE (4.07%) was obtained for the Sb_0.67_Bi_0.33_SI solar cell compared to the SbSI-based cell. However, this method is time-consuming because it requires multiple cycles in step 2 to obtain complete sulfoiodides. In addition, the resulting films were not completely homogeneous. To overcome these limitations, they introduced an SbI_3_ vapor process instead of the SbI_3_ solution process in step 2 (Figure 4c), enabling the production of SbSI with improved homogeneity without repeating step 2 [65] and yielding a better PCE of 3.62% for SbSI solar cells. The study by the Seok group clearly demonstrated a two-step method for fabricating different chalcohalides. However, inherent limitations of the CBD process, such as the formation of impurities and difficulty in controlling the ratio [28,31], may limit the controlled growth of chalcohalides. In addition, factors such as morphology and thickness, which are critical for planar devices, were not considered because the study was optimized for the mesoporous device architecture. Therefore, developing a two-step method allowing the controlled growth of chalcohalides for the planar device architecture remains a challenge.

**Figure 5:**
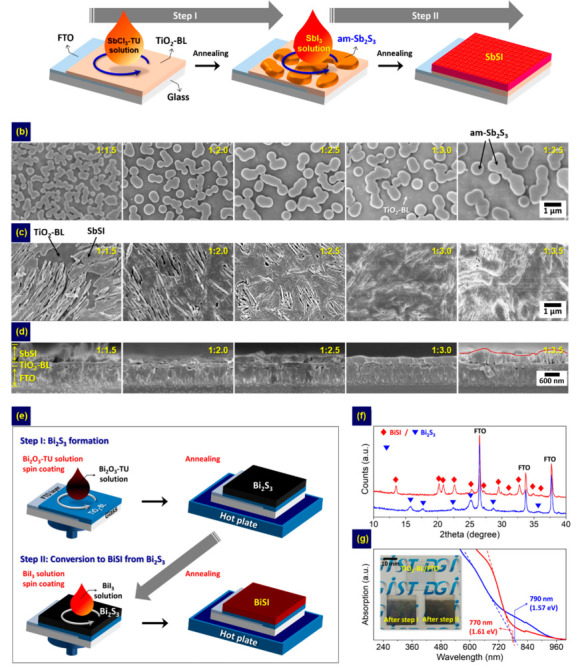
(**a**) Schematic illustration of the two-step method for the SbSI fabrication. Effects of Sb:S ratio on the morphology after: (**b**) step 1; (**c**,**d**) step 2. Adapted under the terms and conditions of the CC BY license [45], copyright 2018, The Authors. Adapted from [45], from AIP Publishing, 2018. (**e**) Schematic diagram of the two-step method for BiSI fabrication. Diagrams showing the (**f**) structure and (**g**) absorption properties of the samples fabricated after step 1 and 2. Adapted under the terms and conditions of the CC BY license [47], copyright 2019, The Authors. Adapted from [47], from MDPI AG, 2019.

**Figure 6:**
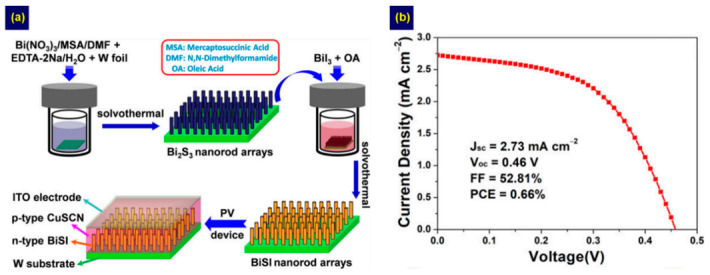
BiSI nanorods array fabrication from Xiong et al. [49] showing: (**a**) a schematic diagram of the BiSI nanorod arrays fabrication procedure and (**b**) a typical current density–voltage curve of *n* ITO/CuSCN/BiSI/W device. Adapted with permission from *ACS Sustainable Chem. Eng.* **2020**, *8*, 13488–13496. Copyright 2020 American Chemical Society [49].

To apply the two-step method to the planar device architecture, a thin film covering the entire surface is necessary. This is because incomplete surface coverage reduces the ability to absorb light and creates the shunt paths, thereby degrading the device performance [66]. We confirmed the feasibility of forming a compact thin film using a two-step method. We introduced an SbCl_3_-TU method instead of the CBD method in step 1 [45], enabling control of the Sb/S ratio and minimizing impurity formation [31]. Then, we used a high-concentration solution to lower the need for multiple cycles in step 2, and this modified method is illustrated in Figure 5a. We found that the Sb/S ratio of the solution used in step 1 significantly affected surface coverage (Figure 5b–d). The annealing conditions of step 2 also contributed to controlling the crystallinity. Then, a compact SbSI thin film with high crystallinity was obtained with an Sb/S specific molar ratio of 1:3 at 200 °C, and an impressive PCE of 0.93% was achieved by the SbSI device. This method allowed us to fabricate pure-phase SbSI thin films and to control morphology and structure. Our method can also be applied for fabricating other chalcohalides such as BiSI [47]. To fabricate BiSI films, we introduced a Bi_2_O_3_-TU solution based on a thiol–amine solvent and BiI_3_ solution in steps 1 and 2, respectively (Figure 5e). Using this method, nanorod-based BiSI films with an *E*g value of 1.61 eV were obtained (Figure 5f,g). Recently, Xiong et al. also reported the fabrication of BiSI nanorods arrays based on a two-step method [49]. However, their method involved the solvothermal synthesis instead of spin coating in each step, as illustrated in Figure 6a. The BiSI nanorods were fabricated by immersing Bi_2_S_3_-deposited tungsten (W) foil in an autoclave containing BiI_3_ solution and subsequent heating. Compared to the spin coating-based two-step process [47], the as-prepared nanorods exhibited a similar *E*g value of 1.57 eV but showed preferential [010] orientation. To fabricate solar cells, a p-type CuSCN and an In-doped SnO_2_ (ITO) were sequentially deposited on the BiSI surface, yielding a PCE of 0.66% (Figure 6b).

The two-step method is also suitable for fabricating the quaternary Pb_2_SbS_2_I_3_. Nie et al. deposited a nanostructured Pb_2_SbS_2_I_3_ with an *E*g of 2.19 eV on an mp-TiO_2_/TiO_2_-BL/FTO substrate [54] for solar cells by modifying the two-step method used for SbSI fabrication [38]. In the modified method, step 1 was identical to that in the SbSI fabrication, whereas a PbI_2_ solution was used in step 2. Through optimization, the best PCE obtained from the Au/PCPDTBT/Pb_2_Sb_2_S_2_I_3_/mp-TiO_2_/TiO_2_-BL/FTO device was 3.2%. These results imply that the two-step method can be simply applied for fabricating Sb/Bi chalcohalides by selecting an appropriate source or reagent in each step.

### 4.3. Other Methods

**Figure 7:**
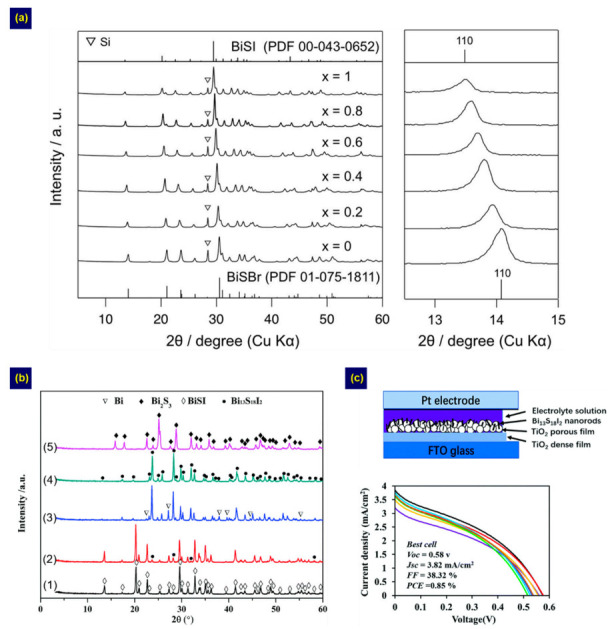
(**a**) Structures of the BiSBr_1−*x*_I*_x_* obtained from BiOBr_1−*x*_I*_x_* under H_2_S gas at 150 °C. Adapted under the terms and conditions of the CC BY license [41], copyright 2016, the authors. Adapted from [41], from Springer Nature, 2016. Structure and device performance for the Bi-S-I compounds synthesized by the solvothermal method: (**b**) Plot showing the effects of the CH_4_N_2_S/BiI_3_/MAI molar ratio including (1) 1:2:3, (2) 2:2:3, (3) 3:2:3, (4) 4:2:3, and (5) 8:2:3 on structures. (**c**) Schematic diagram and *J*–*V* curves of Bi_13_S_18_I_2_ solar cells. Adapted from [53], with permission from Royal Society of Chemistry, 2020.

In addition to the two methods described above, Sb/Bi chalcohalides are fabricated using other approaches. Kunioku et al. reported a low-temperature method based on Bi oxyhalide (BiOX) particles for fabricating Bi chalcohalides (BiChX) [41]. The BiChX were fabricated by substituting Ch^2−^ for the O^2−^ of BiOX particles under H_2_(S,Se) gas. Thus, Bi chalcohalides such as BiSI, BiSeI, BiSSeI, and BiSBr_1−*x*_I*_x_* were obtained by adjusting the starting BiOX and gas type, as shown in Figure 7a. This method enabled BiChX fabrication with controllable *E*g at low temperature (<150 °C). BiSI and BiSeI have also been fabricated by a ball milling method [52]. In addition, one-dimensional SbSI nanostructures were independently manufactured using a mixed sonication–heating method [48] and sonochemical synthesis [50]. Recently, Li et al. fabricated a new type of ternary Bi chalcohalide, the tetragonal Bi_13_S_18_I_2_, in addition to BiSI, with both controlled by adjusting the mole ratio of CH_4_N_2_S/BiI_3_/CH_3_NH_3_I (CH_3_NH_3_I = MAI) in the solution used in the solvothermal process (Figure 7b) [53]. They found that a pure Bi_13_S_18_I_2_ structure can be obtained from the conversion reaction of BiSI over 6 h at a CH_4_N_2_S/BiI_3_/MAI ratio of 4:2:3. The Bi_13_S_18_I_2_ device exhibited a PCE of 0.85% (Figure 7c), demonstrating the potential of Bi_13_S_18_I_2_ as a light absorber for solar cells.

## 5. Sb/Bi Chalcohalides as Interfacial Layer

In addition to being used as light absorbers in solar cells, Sb/Bi chalcohalides can be also used as interfacial layers. Yoo et al. used BiSI as an interlayer in a BiI_3_ solar cell at the interface between the ETL and BiI_3_ light absorber [67]. The BiSI layer was formed in situ on the ETL surface by the reaction of In_2_S_3_ and BiI_3_ at 200 °C during BiI_3_ deposition. The BiSI interlayer greatly improved the hole transfer from BiI_3_ to HTL, improving the PCE to 1.21%. Other chalcohalides can also serve as interlayers. According to the Seok group, the SbSI interlayer formed on the Sb_2_S_3_ surface provides an energetically favorable driving force for photogenerated carriers [65]. Thus, the SbSI-interlayered Sb_2_S_3_ device showed better performance than the Sb_2_S_3_ device, with the best PCE of 6.08%.

## 6. Summary and Outlook

In this review, we summarized the recent progress on the fabrication of Sb/Bi chalcohalide solar cells by focusing on the fabrication methods. Two types of Sb/Bi chalcohalides have been manufactured as Pb-free solar absorbers for solar cells by one-step, two-step, and other methods. The first involves ternary chalcohalides (MChX and M_13_Ch_18_X_2_), while the other comprises quaternary chalcohalides ($M_{2}^{\mathrm{II}}M^{\mathrm{III}}{\mathrm{Ch}}_{2}X_{3}$). Maximum PCEs of 4.07% and 4.04% were obtained from the ternary Sb_0.67_Bi_0.33_SI and quaternary Sn_2_SbS_2_I_3_ solar cells, respectively. In addition, ternary BiSI and SbSI acted as interfacial layers in solar cells, contributing to enhanced charge transfer. Although Sb/Bi chalcohalides with excellent stability have been proposed over the past few years, their PCEs still significantly lag behind those of Pb-perovskites. Therefore, an in-depth comprehensive investigation into the intrinsic and extrinsic factors affecting device performance is required. The impact of material composition, morphology, device architecture, crystal orientation, and interfacial layer, as well as the factors affecting performance degradation and device stability, also require detailed examination to further improve the performance of devices [61,66,68].

## Figures and Tables

**Table 1:** Types of Pb-free photovoltaic materials and their best photovoltaic performance data.

	Metal (M) Ions	Chemical Compound	Record Device Performance		
			PCE	Material	Ref.
Perovskites	Sn^2+^, Ge^2+^	Perovskite/AMX_3_	11.4%	FASnI_3_	[18]
	Ag^+^, Bi^3+^	Double perovskite/A_2_M^I^M^III^X_6_	2.84%	Cs_2_AgBiBr_6_	[19]
	Sn^4+^	Vacancy-ordered double perovskite/A_2_M^IV^X_6_	3.28%	Cs_2_TiBr_6_	[20]
	Sb^3+^, Bi^3+^	2D perovskite/$A_{3}M_{2}^{\mathrm{III}}X_{9}$	3.34%	MA_3_Sb_2_I_9−*x*_Cl*_x_*	[21]
Sb/Bi-based non-perovskites	Sb^3+^	Sb chalcogenides/M_2_Ch_3,_ CuMCh_2_	10.5%	Sb_2_(S,Se)_3_	[22]
	Sb^3+^, Bi^3+^	Ternary chalcohalides/MChX, M_13_Ch_18_X_2_	4.07%	Sb_0.67_Bi_0.33_SI	[23]
	Sn^2+^, Pb^2+^, Sb^3+^, Bi^3+^	Quaternary chalcohalides/$M_{2}^{\mathrm{II}}M^{\mathrm{III}}{\mathrm{Ch}}_{2}X_{3}$	4.04%	Sn_2_SbS_2_I_3_	[24]

PCE—power conversion efficiency.

**Table 2:** Summarized data for the structural properties of Sb/Bi chalcohalides used for solar cells.

	Chemical Formula	Structure/Space Group	Typical Materials	Ref.
Ternary chalcohalides	MChX	Orthorhombic/*Pnma*	SbSI, BiSI	[23,38,39,40,41,42,43,44,45,46,47,49,58]
	M_13_Ch_18_X_2_	Hexagonal/*P6_3_*	Bi_13_S_18_I_2_	[53,58]
Quaternary chalcohalide	$M_{2}^{\mathrm{II}}M^{\mathrm{III}}{\mathrm{Ch}}_{2}X_{3}$	Orthorhombic/*Cmcm*	Pb_2_SbS_2_I_3_, Sn_2_SbS_2_I_3_	[24,54,59]

**Table 3:** Summary of effective masses of hole (*m*_h_*) and electron (*m*_e_*), static dielectric constant, and absorption coefficient of MChX family, calculated by different methods.

MChX Compounds	*m*_h_*	*m*_e_*	Static Dielectric Constant	Absorption Coefficient ^1^	References
Pb-perovskite ^2^	0.10	0.16	20.07	>1 × 10^5^ cm^−1^	[61,62]
BiSI	0.61–4.79	0.53–2.33	14.26–71.32	>1 × 10^5^ cm^−1^	[39,44,62,63]
BiSeI	0.81–5.89	0.25–1.61	14.78–62.82	>1 × 10^5^ cm^−1^	[39,44,62,63]
SbSI	0.27–2.06	0.21–1.25	10.56–69.38	-	[42,55,62,63,64]
SbSeI	0.57–4.37	0.35–1.83	14.70–57.18	-	[42,55,62,63,64]
SbSBr	0.24–3.55	0.51, 0.52	13.81–105.15	-	[42,55,63,64]

^1^ Absorption coefficient values at visible region are presented. ^2^ Data of (CH_3_NH_3_)PbI_3_ are shown as typical of Pb-perovskites for comparison.

**Table 4:** Summary of Sb/Bi chalcohalides fabricated for solar cells using varied methods.

Method	Chalcohalide	Device Structure	PCE (%)/J_SC_^1^ (mA·cm^−2^)/V_OC_^2^ (V)/FF ^3^	Ref.
One-step deposition	Bi(S,Se)I	FTO/Pt/CuSCN/Bi(S,Se)I/FTO	0.01/0.07/0.39/0.4	[40]
	BiSI	Au/F8/BiSI/SnO_2_/FTO	1.32/8.44/0.45/0.35	[46]
	SbSI	Au/PEDOT:PSS ^4^/PCPDTBT/Sb_2_S_3_-SbSI/mp-TiO_2_/TiO_2_-BL/FTO	2.91/12.0/0.47/0.52	[51]
	Sn_2_SbS_2_I_3_	Au/PCPDTBT/Sn_2_SbS_2_I_3_/mp-TiO_2_/TiO_2_-BL/FTO	4.04/16.1/0.44/0.57	[24]
Two-step deposition	SbSI	Au/PCPDTBT/SbSI/mp-TiO_2_/TiO_2_-BL/FTO	3.05/9.11/0.58/0.58	[38]
	Sb_0.67_Bi_0.33_SI	Au/PEDOT:PSS/PCPDTBT/Sb_0.67_Bi_0.33_SI/mp-TiO_2_/TiO_2_-BL/FTO	4.07/14.54/0.53/0.53	[23]
	SbSI	Au/PCPDTBT/SbSI/mp-TiO_2_/TiO_2_-BL/FTO	3.62/9.26/0.6 /0.65	[65]
	SbSI	Au/P3HT/SbSI/TiO_2_-BL/FTO	0.93/5.45/0.55/0.31	[45]
	BiSI	Au/P3HT/BiSI/TiO_2_-BL/FTO	-	[47]
	BiSI	ITO/CuSCN/BiSI/W	0.66/2.73/0.46/0.53	[49]
	Pb_2_SbS_2_I_3_	Au/PCPDTBT/Pb_2_SbS_2_I_3_/mp-TiO_2_/TiO_2_-BL/FTO	3.12/8.79/0.61/0.58	[54]
Oxyhalides conversion	Bi(S,Se)(I,Br)	No device	-	[41]
Mixed sonication-heating	SbSI	Carbon/ZrO_2_/SbSI/mp-TiO_2_/TiO_2_-BL/FTO	0.04/0.05/0.29/0.31	[48]
Sonochemical method	SbSI	Au/P3HT/SbSI-PAN/TiO_2_ NP/ITO	-	[50]
Solvothermal method	Bi_13_S_18_I_2_	Pt/Electrolyte/Bi_13_S_18_I_2_/mp-TiO_2_/TiO_2_-BL/FTO	0.85/3.82/0.58/0.38	[53]

^1^ J_SC_, ^2^ V_OC_, ^3^ FF, and ^4^ PEDOT:PSS indicate short-circuit current density, open-circuit voltage, fill factor, and poly(3,4-ethylenedioxythiophene):poly(styrene sulfonate), respectively; BL—blocking layer.
